# A novel approach to detect KRAS/BRAF mutation for colon cancer: Highly sensitive simultaneous detection of mutations and simple pre-treatment without DNA extraction

**DOI:** 10.3892/ijo.2015.2978

**Published:** 2015-04-30

**Authors:** SHUN-ICHI SUZUKI, SATOSHI MATSUSAKA, MITSUHARU HIRAI, HARUMI SHIBATA, KOICHI TAKAGI, NOBUYUKI MIZUNUMA, KIYOHIKO HATAKE

**Affiliations:** 1 ARKRAY Marketing Inc., Marketing Division, Shijuku-ku, Tokyo 160-0004, Japan; 2 Gastroenterological Center, Cancer Institute Hospital of Japanese Foundation for Cancer Research, Koto-ku, Tokyo 135-8550, Japan; 3 ARKRAY Inc., Research and Development Division, Kamigyou-ku, Kyoto 602-0008, Japan; 4 Cancer Chemotherapy Center, Clinical Chemotherapy of Japanese Foundation for Cancer Research, Koto-ku, Tokyo 135-8550, Japan

**Keywords:** colon cancer, KRAS mutation, BRAF mutation, QProbe method

## Abstract

It has been reported that colon cancer patients with KRAS and BRAF mutations that lie downstream of epidermal growth factor receptor (EGFR) acquire resistance against therapy with anti-EGFR antibodies, cetuximab and panitumumab. On the other hand, some reports say KRAS codon 13 mutation (p.G13D) has lower resistance against anti-EGFR antibodies, thus there is a substantial need for detection of specific KRAS mutations. We have established a state-of-theart measurement system using QProbe (QP) method that allows simultaneous measurement of KRAS codon 12/13, p.G13D and BRAF mutation, and compared this method against Direct Sequencing (DS) using 182 specimens from colon cancer patients. In addition, 32 biopsy specimens were processed with a novel pre-treatment method without DNA purification in order to detect KRAS/BRAF. As a result of KRAS mutation measurement, concordance rate between the QP method and DS method was 81.4% (144/177) except for the 5 specimens that were undeterminable. Among them, 29 specimens became positive with QP method and negative with DS method. BRAF was measured with QP method only, and the mutation detection rate was 3.9% (6/153). KRAS measurement using a simple new pre-treatment method without DNA extraction resulted in 31 good results out of 32, all of them matching with the DS method. We have established a simple but highly sensitive simultaneous detection system for KRAS/BRAF. Moreover, introduction of the novel pre-treatment technology eliminated the inconvenient DNA extraction process. From this research achievement, we not only anticipate quick and accurate results returned in the clinical field but also contribution in improving the test quality and work efficiency.

## Introduction

In recent years, research on the relation between genetic mutation and cancer treatment efficiency is making progress, which is being applied to the development of new drugs, especially molecular target drugs. Along with the popularization of molecular target drugs, diagnosis before medication has been growing rapidly in practical use for drug selection and/or decision making on the treatment strategy (1–3). There are many types of molecular target drugs to target epidermal growth factor receptor (EGFR) such as tyrosine kinase inhibitor; a low-molecular compound, and antibody drugs. Oncogenic mutation that lies downstream of EGFR target drugs is a signal transduction molecule, and it is extremely important to check this mutation for predicting drug efficacy (4). Anti-EGFR antibody, cetuximab or panitumumab is a treatment for colon cancer that is highly effective to patients with expression of EGFR protein; however, it has been reported that patients with KRAS gene mutations that lie downstream acquire resistance against therapy (5–7).

KRAS is a signal transduction molecule that is playing a part in mitogen-activated protein kinase (MAPK) pathway that lies downstream of EGFR and is related to cell proliferation and angiogenesis (8). KRAS gene mutation is known for inducing constitutive activation of KRAS and stimulating cancer growth, and it is found in various organs, such as the colon, pancreas and lungs. In Japanese population, KRAS gene mutation is found in 30–42% of colon cancer patients (9). The mutations are found mainly in codons 12 and 13. Due to single nucleotide or dinucleotide mutations, some amino acids are substituted with other amino acids. It is known that resistance against anti-EGFR antibodies will be acquired when there is a mutation in KRAS codon 12/13. However, there is an interesting report that mutation in codon 13 (p.G13D) has lower resistance against anti-EGFR antibodies compared with other mutations and extends the overall survival and progression-free survival time of the patient (10,11). Thus, there is a high possibility that detection of p.G13D apart from other mutations will have clinical importance in the future.

The entire drug efficacy cannot be predicted just from KRAS gene mutation itself and other factors are likely be involved. One of the factors is the BRAF gene mutation (V600E) that lies downstream of EGFR, similarly to KRAS (9). BRAF V600E mutation has been found in ~4.7% of the colon cancer patients in Japan. Again, similarly to KRAS mutation, constant self-activation is considered to induce the activity of signal pathway and stimulate canceration (9). There has also been a report that it has resistance against treatment with anti-EGFR antibodies (12). It is invaluable to give appropriate therapeutic opportunity to patients to whom treatment will be effective. Thus, checking these items for diagnosis prior to drug administration is highly beneficial.

In order to spread these genetic mutation tests with great clinical significance, we have established a measurement system that allows simultaneous measurements of KRAS codon 12/13, p.G13D and BRAF. This system uses QProbe (QP) method and can detect these mutations quickly and relatively easily. In this study, the accuracy of the novel system was compared to that of the conventional Direct Sequence (DS) method. For gene analysis, we have also studied a revolutionary pre-treatment that does not require any complicated operations such as DNA purification, which is also reported herein.

## Materials and methods

### Specimens

Tissues collected from 182 colon cancer patients who received surgery between 2009 and 2010 at The Cancer Institute Hospital of JFCR were used as specimens. Purified DNA was extracted from the specimens using QIAamp DNA FFPE Tissue kit (Qiagen, Hilden, Germany). Also, frozen biopsy specimens (32 specimens) collected at the same hospital in 2011 were used for measurement without extracting DNA. All the patients enrolled in this study were approved by the Institutional Review Board at the Cancer Institute Hospital of the Japanese Foundation for Cancer Research (JFCR). Written informed consent was obtained from each patient.

### Detection of KRAS mutation

KRAS mutation was detected using the DS method and QP method (13). For DS method based on nested polymerase chain reaction (PCR), the primer sequences listed below and PCR reaction conditions were used in the measurement.

Primers used in the first round PCR reaction were 5′-ggagtatttgatagtgtattaacct-3′ (sense) and 5′-gaaaatggtcagagaaacc tttatc-3′ (antisense). Conditions of PCR reaction were a cycle of 1 min at 94°C, 10 sec at 98°C, 5 sec at 55°C and 30 sec at 72°C, repeated 40 times. After reaction, they were stored at 4°C. For the second round PCR reaction, primers 5′-gtgtgacatgttctaatatagtca-3′ (sense) and 5′-gtcctgcaccagtaatatgc-3′ (antisense) were used. Conditions of PCR reaction were a cycle of 1 min at 94°C, 10 sec at 98°C, 15 sec at 55°C and 30 sec at 72°C, repeated 30 times. Reagent used for PCR reaction was Prime STAR HS (Premix) (Takara Bio Inc., Shiga, Japan) and instrument was GeneAmp PCR System 9700 (Applied Biosystems, Foster City, CA, USA). Amplified product was purified using QIAquik PCR Purification kit (Qiagen). Sequencing reaction was performed using BigDye Terminator v3.1 Cycle Sequencing kit (Applied Biosystems), and primers of the same sort used in the second round PCR reaction were used for each reaction. Then, they were purified using BigDye XTerminator Purification kit (Applied Biosystems) for checking the sequence information with Applied Biosystems 3130x/Genetic Analyzer (Applied Biosystems).

The following primer and QProbe sequences were used for QP method measurement: primer sequences for detecting KRAS mutations are 5′-aaggcctgctgaaaatgactg-3′ (sense) and 5′-ggtcctgcaccagtaatatgca-3′ (antisense). QProbe (Nippon Steel & Sumikin Eco-Tech Corp., Tokyo, Japan) sequences are 5′-(BODIPY FL)-ctcttgcctacgccaccagctccaact-3′ for detecting KRAS codon 12/13 and 5′-(Pacific Blue)-cttgcc tacgtca-3′ for detecting p.G13D. In QP method, existence of KRAS codon 12/13 mutation can be detected; however, codon 12 and 13 cannot be distinguished since probes are designed for wild-type of KRAS codon 12/13 (14,15). However, G13D can be distinguished by matching specific probes with p.G13D.

Moreover, KRAS and BRAF mutation were detected simultaneously in a single measurement by combining primers and probes for BRAF V600E mutation detection. The following primer and QProbe sequences were used for measuring BRAF mutation:

Primer sequence for detecting BRAF V600E mutation is 5′-tgcttgctctgataggaaaatgagatctac-3′ (sense) and 5′-aaact gatgggacccactccat-3′ (antisense). QProbe sequence is 5′-gctaca gAgaaatctc-(TAMRA)-3′. For the QP method, PCR and detection processes were performed automatically using fully automated gene analyzer, i-densy™ IS-5320 (Arkray Inc., Kyoto, Japan). Before the measurement, the necessary number of tips, reaction tubes, reagent packs and specimens are set in the instrument. The results become available in 90 min just by pressing the START button (16). Since up to three mutations can be run in parallel with a single reagent pack, we have tested the KRAS codon 12/13, p.G13D and BRAF V600E mutations all at once. After 1 min of initial degeneration at 95°C, i-densy™ repeats 60 cycles of PCR: heat degeneration for 1 se at 95°C and annealing for 30 sec at 62°C. After the completion of PCR, mutation is detected through Tm analysis. Mutations are identified by the differences in melting temperature. Specimens that gave different results in DS method and QP method were checked by the Scorpion-ARMS method.

### Simple pre-treatment without DNA extraction

Biopsy specimen (≤1-mm^3^) was diluted to 10 μl with purified water and heat processed for 5 min at 95°C. When biopsy specimen was large, it was lightly homogenized to become <1-mm^3^. This method was used for the PCR reactions and detections described below.

## Results

### Detection of KRAS mutations using the DS and QP methods

DNA extracted from tissues collected from 182 colon cancer patients was used to check the existence of KRAS mutation (Table I). As a result, DS method screened 121 cases out of 182 as KRAS mutation-negative, 47 cases as KRAS codon 12 mutation-positive and 14 cases as KRAS codon 13 (p.G13D)-positive. On the other hand, QP method screened 92 cases out of 182 as KRAS mutation-negative, 72 cases as KRAS codon 12 mutation-positive, 13 cases as p.G13D-positive and 5 cases as undeterminable. The concordance rate of the two methods regarding KRAS-negative, codon 12-positive and p.G13D-positive results were 75.2, 93.6 and 92.3%, respectively, and 81.4% on average (excluding undeterminable specimens). There were as many as 33 specimens with diverging results. Putting aside the 5 undeterminable cases with QP method, among the 117 cases that became KRAS mutation-negative with DS method, specimens with diverging results from QP method were as many as 29 cases (28 cases for codon 12 mutation-positive and one case of p.G13D-positive). In addition, 153 cases of BRAF V600E mutation were also measured in parallel with KRAS mutation. Although the measurement was only done with the QP method, 6 cases became positive and the frequency of occurrence was 3.9% (data not shown).

### QP method has higher capacity for KRAS mutation detection compared to the DS method

Among 33 specimens with diverging results between DS method and QP method, 12 specimens could be retested (Table II). In order to search for the cause of the divergence, 12 specimens were retested with Scorpion-ARMS method (Table III). As a result, among the 10 cases where codon 12 mutation became negative with DS method and positive with QP method, 7 cases became positive with Scorpion-ARMS method. Also, 2 cases where the results were negative with DS method became negative with both QP method and Scorpion-ARMS method.

**Figure 1:**
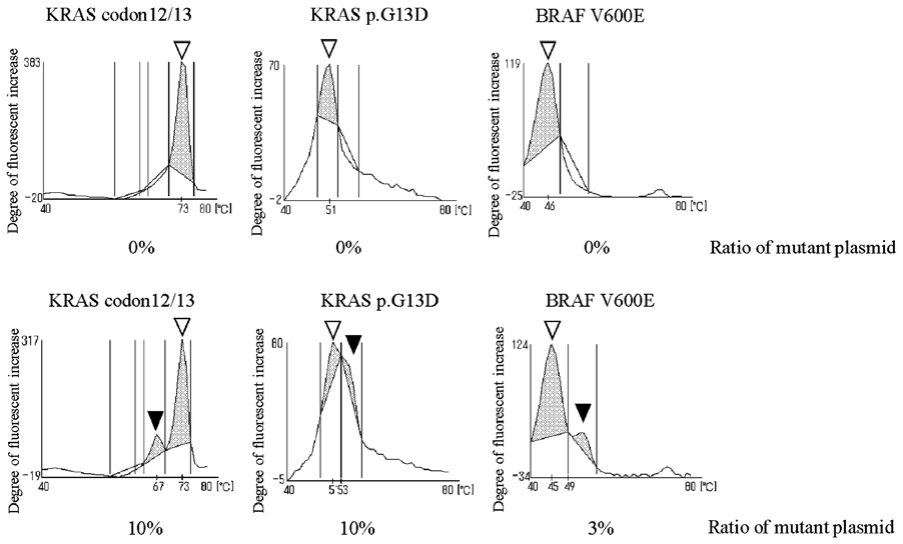
Detection of KRAS and BRAF mutation using the QP method. Detection limit of the QP method using a control plasmid containing the KRAS mutation, codon 12/13(GGTGGC→GATGGC) or p.G13D(GGTGGC→GGTGAC) and BRAF V600E mutation. Wild-type (wt) peak (▽) and mutant (mt) peak (▼) are shown.

The QP method determines the existence of mutation by the existence of peaks. In this system, clear peaks were found in specimens with mutation plasmid content at 10% for KRAS codon 12/13 mutation, 10% for p.G13D and 3% for BRAF V600E mutation (Fig. 1). A peak area can be calculated by specifying the temperature range where the peak appears (Table IV). In this evaluation, cut-off values were set at a range where a clear mutation peak can be obtained (KRAS codon 12/13: mt 10%, BRAF V600E: mt 3%) in order to prevent false detection (Table V). Plasmid is being used for the criteria of both the DS method and QP method. The results of the DS method are considered to be positive when the mutation base waveforms are confirmed by visual inspection in both sense and antisense results. The detection sensitivity of KRAS codon 12/13 mutation using the DS method is estimated to be ~10%. From the above, KRAS mutation detectability with QP method in this evaluation using the actual sample is better than the DS method and is equivalent to or better than Scorpion-ARMS method that have relatively high sensitivity.

### KRAS mutation can be detected directly from specimen tissue without DNA extraction

**Figure 2:**
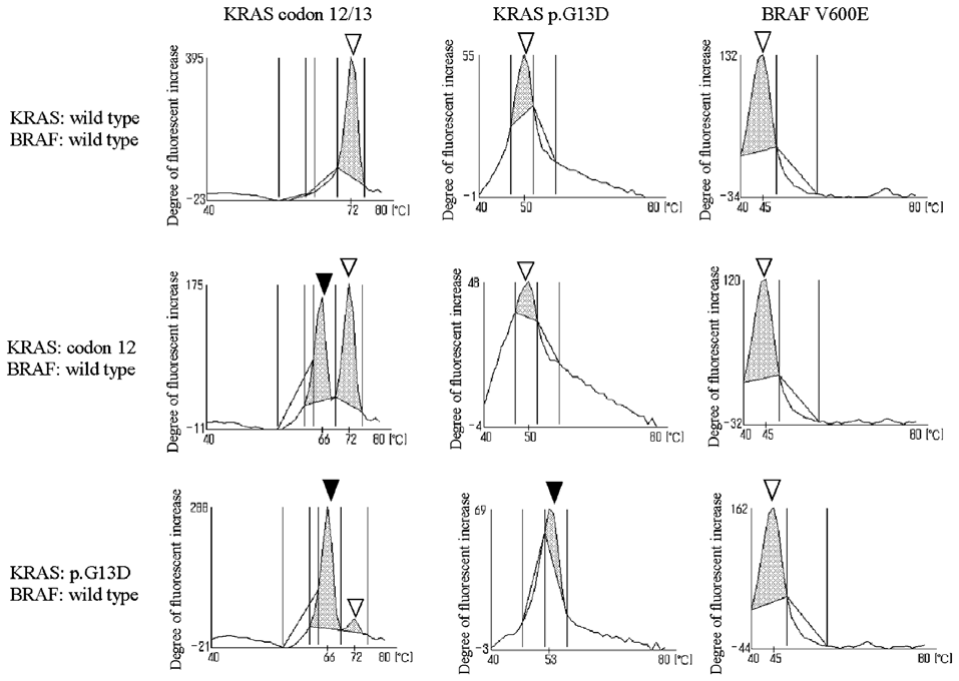
Detection of KRAS and BRAF mutation using the QP method after a novel pretreatment protocol that omits purification process of DNA. Major example of each result (KRAS: wild-type/BRAF: wild-type 18 cases, KRAS: codon 12/BRAF: wild-type 10 cases, KRAS: p.G13D/BRAF: wild-type 3 cases) are indicated.

Gene analyzer i-densy IS-5320 uses QP method and can analyze genes from whole blood in full automation by using special reagents. In this evaluation, a new protocol was evaluated with 32 biopsy specimens collected from colon cancer patients, which was heated at 95°C by i-densy as pretreatment and used for gene analysis. Biopsy specimens of 1-mm^3^ were used. KRAS mutation in all of these specimens was analyzed from purified DNA in advance by Luminex method. As a result of testing with i-densy, good reaction was seen in 31 out of 32 cases in perfect match with Luminex method (Table VI). Specimens that gave results had clear peaks, and most of the mt peaks were evidently higher than wt peaks (Fig. 2). False reaction was seen in one case, and the possible cause of this was the large biopsy specimen (~3 mm^3^) causing insufficient homogenization before heat treatment. Since Multiplex reagent was used for this measurement, BRAF V600E mutation was tested at the same time, and all 32 cases gave negative results.

## Discussion

In recent years, development of molecular target drugs for cancer treatment is making progress, and gene test before drug administration is becoming indispensable. Anti-EGFR antibody is a drug targeting EGFR and is widely applied in the clinical field. There have been many reports on the acquisition of drug resistance when there is a mutation in KRAS codon 12/13 or BRAF V600 that lie downstream of EGFR (17–20). On the other hand, anti-EGFR antibodies may have drug efficacy for p.G13D mutation among KRAS codon 12/13 mutation, so it will be useful in the future to determine the existence of KRAS mutation by distinguishing this mutation (10,11). In this study, we have established a measurement system that can detect the KRAS codon 12/13, p.G13D and BRAF V600E at the same time. Compared to the system introduced in the previous studies (14,15), the new system has improved detection sensitivity and capability to identify p.G13D. Using QP method, this system made it possible to detect these mutations in high sensitivity compared to DS method based on nested PCR. Moreover, the use of gene analyzer i-densy led to the successful gene analysis from specimen tissue just with simple pre-treatment without going through troublesome DNA extraction.

This system was compared with the conventional DS method to check its accuracy, and divergence was seen in 33 out of 177 cases (Tables I and II). In particular, as many as 29 specimens became negative with DS method, but positive with the QP method. Among 10 positive specimens with QP method that became negative with DS method, 7 out of 10 became positive with Scorpion-ARMS method, which suggest a high possibility that DS method is overlooking KRAS mutation (Table III). Although QP and DS methods were equivalent in mutation detection test using plasmid, many specimens with inconsistent results were found in this study, which leads to the following two causes to be considered. i) Difference in copy-count sensitivity: QP method can amplify and detect plasmid with only two copies, which means the copy-count sensitivity is extremely high (data not shown). Thus, this method can measure DNA with mutation even if the absolute quantity is very small. For example, even if the content rate of DNA mutation is >10%, pathological tissue specimen of poor quality will not be detected with DS method if PCR cannot be amplified up to the volume required to detect DNA quantity. Even if the results are equivalent in mutation detectivity test with sufficient copies of plasmid (8,000 copies), the results may be inconsistent when testing actual samples due to the possible shortage of copy-count. ii) Wild-type inhibition system of QP method: KRAS measurement series of QP method have the probes designed to be in perfect match with wild-type and mismatch with mutant type. Wild inhibition system can intentionally amplify mutant type sequence by adjusting the probe sequence and temperature conditions to inhibit amplification of wild-type sequence during PCR. The mutation detection of this system improves under the condition of low enzyme activity where PCR efficiency decreases. Unlike plasmid, the actual samples contain foreign substances such as protein, which reduce the PCR efficiency. Moreover, when samples contain DNA in advanced fragmentation stage due to formalin fixation, fragmented DNA is assumed to cause PCR inhibition as a foreign substance. Thus PCR efficiency is expected to drop compared to tests using plasmid, resulting in a possibility of mutation detection to be better than 10%, which is the expected sensitivity of this system. Furthermore, 2 specimens that were positive with DS method became negative with the other two methods. Therefore, contamination from DS method technique and erroneous decisions were called into question. Although DS method is a proven method with stable results, there are a few risks due to the manual work involved, leading to contamination or mix-up of specimens. Moreover, the decision is made by visual inspection, so there could be a possibility of mistaking a noise waveform for a small mutation waveform. On the other hand, QP method and Scorpion-ARMS method have clear criteria and less manual work involved before the measurement, reducing such risk. These results indicate that the system we have developed offers highly sensitive and accurate gene analysis using DS method with sensitivity equivalent to or better than Scorpion-ARMS method.

BRAF V600E mutation was measured only with the QP method, and the frequency of occurrence was 3.9% in this study. The lower detection limit of BRAF V600E mutation was 3%, adjusting mutant plasmid to this level. This measurement system clearly had higher sensitivity compared to the DS method. Moreover, this test result matched with the general report that the frequency of BRAF mutation occurrence in Japanese colon cancer patients is ~4–5% (9). As of now, definite treatment is not established for BRAF mutation-positive cases, and the primary goal of the measurement is to avoid unnecessary administration of anti-EGFR antibody drug. Vemurafenib is an effective treatment drug for malignant melanoma that is BRAF mutation-positive; however, it is known to have little effect on BRAF mutation-positive colon cancer. Activation of EGFR is known to be a mechanism of vemurafenib-resistance (21). Therefore, there is a high degree of expectation in combination therapy of BRAF inhibitor and anti-EGFR antigen, etc. for BRAF mutation-positive colon cancer, and the outcome of future clinical trials is eagerly expected. As indicated above, studies for new treatment methods are advancing. They will gain recognition in the future as screening test items before drug administration for colon cancer treatment along with BRAF and KRAS.

Purified DNA is typically used for gene analysis; however, we have tried eliminating DNA purification process to perform gene analysis with simple pre-treatment. Thirty-two frozen biopsy specimens were measured with simple pre-treatment without extracting DNA. All 31 cases that gave results fully matched with the results of Luminex method (Table VI). Since special reagent for i-densy used for the analysis was developed for the measurement from whole blood, it is not prone to being affected by foreign substances such as protein. There have been many reports on gaining good test results from whole blood (22–25). The special quality of this reagent was most likely the major reason for good results even without DNA purification from sample tissue. In addition, all 12 specimens measured directly from the tissue that became KRAS mutation-positive could be clearly identified as positive because the mutation peaks were equivalent to or larger than that of wild-type. This is likely to be caused by the high content rate of cancer cells in biopsy specimen used for measurement because the tissues are collected with endoscope directly from cancer tissues. There was one PCR failure out of 32 cases; however, this is presumed to be caused by excess sample volume. Although the reagent composition is robust over foreign substances, sample volume must be controlled strictly in order to secure good results. When using formalin fixation tissues, it is known that tissue amount >1 mm^3^ will likely cause reaction failure, and all reactions with tissue amount >2 mm^3^ will result in a failure. Tissue specimens of ≤1 mm would be sufficient for good analysis results. When the sample volume exceeds this level, it is desirable to adjust the sample volume by homogenizing before use. Although existence of cancer cells was not examined by pathologic testing for this study, it is desirable to check for cancer cells for instance by hematoxylin and eosin stain before genetic testing in order to improve mutation detectivity. However, obtaining a large tissue from an elderly or patient with metastatic cancer would be difficult. The fact that genetic testing is possible by using micro amount of specimen collected with an endoscope is one of the merits of this method. Since the sample volume required for the genetic testing is very small, sufficient amount of specimens can be sent for pathologic testing. Moreover, deparaffinization of paraffin-embedded tissues (FFPE) allows measurements to be performed with the same protocol as frozen tissues, and liquid cytological specimens can be used for measurement, making this method widely applicable to examinations of micro amount of specimens. This pre-treatment protocol allows eliminating DNA purification process that typically takes place and performing simple, quick and accurate gene analysis. By using this protocol, complicated operations, labor time and reagent costs can be reduced.

In order to predict the drug efficacy of molecular target medicine targeting EGFR, it is important to check the mutation of KRAS and BRAF genes, which are the signals that lie downstream of EGFR. In addition, p.G13D among KRAS codon 12/13 mutations may likely have a different drug efficacy compared to other mutations, thus further clinical validation would be desirable. Today, there is not enough evidence supporting the drug efficacy of p.G13D, which is why it is not actively considered at the time of drug administration. In a case of KRAS p.G13D-positive, FOLFOX or FOLFIRI ± bevacizumab treatment is administered in 1st and 2nd lines, and chemotherapy such as regorafenib or TAS-102 monotherapy are considered for the 3rd and 4th lines (26,27). However, when the treatment reaches a stage beyond that point, anti-EGFR antibodies may be selected, thus it is helpful to identify p.G13D beforehand.

Measurement using i-densy with QP method allows quick and simple detection of gene mutations simultaneously from a single reaction system, with sensitivity better than DS method. Furthermore, innovative and simplified pre-treatment protocol reduces the operation process and realizes simplified and accurate gene analysis that can be performed by any user. By changing PCR primer and QProbe, this versatile instrument can be used to detect other gene mutations that may affect the efficacy of molecular target treatment. Recently, NRAS has been reported as a drug-resistant mutation similar to KRAS, which detecting system needs to be established soon (28–30). We sincerely hope that this study will drive the gene analysis to spread to further clinical sites and to contribute to personalized medicine in the future.

## Figures and Tables

**Table I:** Comparison of KRAS codon 12/13 mutation between the DS and QP methods.

	QProbe method				
			
KRAS mutation^a^	Wild-type	Codon 12	p.G13D	Undeterminable	Concordance rate^c^
Wild-type	88	28	1	4	72.7% (75.2%)
Codon 12^b^	3	44	0	0	93.6%
p.G13D	1	0	12	1	85.7% (92.3%)
					Total 79.1% (81.4 %)

a Results from Direct Sequence method.

b Codon 12/13 mutations except p.G13D.

c Numbers in parenthesis exclude samples with no results.

**Table II:** Comparison of KRAS codon 12/13 mutation between the DS and QP methods.

No.	DS	QProbe
1	p.G12N	Codon 12
2	p.G12V	Codon 12
3	p.G12V	Codon 12
4	p.G12V	Codon 12
5	p.G12D	Codon 12
6	p.G12D	Codon 12
7	p.G12D	Codon 12
8	p.G12V	Codon 12
9	p.G12D	Codon 12
10	p.G12D	Codon 12
11	p.G12D	Codon 12
12	p.G12D	Codon 12
13	p.G12S	Codon 12
14	p.G12D	Codon 12
15	p.G12D	Codon 12
16	p.G12S	Codon 12
17	p.G12D	Codon 12
18	p.G12D	Codon 12
19	p.G12D	Codon 12
20	p.G12D	Codon 12
21	p.G12D	Codon 12
22	p.G12V	Codon 12
23	p.G12V	Codon 12
24	p.G12S	Codon 12
25	p.G12S	Codon 12
26	p.G12D	Codon 12
27	p.G12C	Codon 12
28	p.G12D	Codon 12
29	p.G12S	Codon 12
30	p.G12D	Codon 12
31	p.G12C	Codon 12
32	p.G12V	Codon 12
33	p.G12D	Codon 12
34	p.G12D	Codon 12
35	p.G12D	Codon 12
36	p.G12V	Codon 12
37	p.G12D	Codon 12
38	p.G12D	Codon 12
39	p.G12D	Codon 12
40	p.G12V	Codon 12
41	p.G12D	Codon 12
42	p.G12D	Codon 12
43	p.G12S	Codon 12
44	p.G12A	Codon 12
45	p.G12V/D	Wild-type
46^a^	p.G12D	Wild-type
47	p.G12D	Wild-type
48	p.G13D	p.G13D
49	p.G13D	p.G13D
50	p.G13D	p.G13D
51	p.G13D	p.G13D
52	p.G13D	p.G13D
53	p.G13D	p.G13D
54	p.G13D	p.G13D
55	p.G13D	p.G13D
56	p.G13D	p.G13D
57	p.G13D	p.G13D
58	p.G13D	p.G13D
59	p.G13D	p.G13D
60^a^	p.G13D	Wild-type
61	p.G13D	-
62	Wild-type	p.G13D
63^a^	Wild-type	Codon 12
64^a^	Wild-type	Codon 12
65^a^	Wild-type	Codon 12
66	Wild-type	Codon 12
67	Wild-type	Codon 12
68	Wild-type	Codon 12
69^a^	Wild-type	Codon 12
70	Wild-type	Codon 12
71	Wild-type	Codon 12
72^a^	Wild-type	Codon 12
73^a^	Wild-type	Codon 12
74	Wild-type	Codon 12
75	Wild-type	Codon 12
76	Wild-type	Codon 12
77	Wild-type	Codon 12
78	Wild-type	Codon 12
79^a^	Wild-type	Codon 12
80^a^	Wild-type	Codon 12
81^a^	Wild-type	Codon 12
82^a^	Wild-type	Codon 12
83	Wild-type	Codon 12
84	Wild-type	Codon 12
85	Wild-type	Codon 12
86	Wild-type	Codon 12
87	Wild-type	Codon 12
88	Wild-type	Codon 12
89	Wild-type	Codon 12
90	Wild-type	Codon 12
91	Wild-type	Wild-type
92	Wild-type	Wild-type
93	Wild-type	Wild-type
94	Wild-type	Wild-type
95	Wild-type	Wild-type
96	Wild-type	Wild-type
97	Wild-type	Wild-type
98	Wild-type	Wild-type
99	Wild-type	Wild-type
100	Wild-type	Wild-type
101	Wild-type	Wild-type
102	Wild-type	Wild-type
103	Wild-type	Wild-type
104	Wild-type	-
105	Wild-type	Wild-type
106	Wild-type	Wild-type
107	Wild-type	Wild-type
108	Wild-type	Wild-type
109	Wild-type	-
110	Wild-type	Wild-type
111	Wild-type	Wild-type
112	Wild-type	Wild-type
113	Wild-type	Wild-type
114	Wild-type	Wild-type
115	Wild-type	Wild-type
116	Wild-type	Wild-type
117	Wild-type	Wild-type
118	Wild-type	Wild-type
119	Wild-type	-
120	Wild-type	Wild-type
121	Wild-type	Wild-type
122	Wild-type	
123	Wild-type	Wild-type
124	Wild-type	Wild-type
125	Wild-type	Wild-type
126	Wild-type	Wild-type
127	Wild-type	Wild-type
128	Wild-type	Wild-type
129	Wild-type	Wild-type
130	Wild-type	Wild-type
131	Wild-type	Wild-type
132	Wild-type	Wild-type
133	Wild-type	Wild-type
134	Wild-type	Wild-type
135	Wild-type	Wild-type
136	Wild-type	Wild-type
137	Wild-type	Wild-type
138	Wild-type	Wild-type
139	Wild-type	Wild-type
140	Wild-type	Wild-type
141	Wild-type	Wild-type
142	Wild-type	Wild-type
143	Wild-type	Wild-type
144	Wild-type	Wild-type
145	Wild-type	Wild-type
146	Wild-type	Wild-type
147	Wild-type	Wild-type
148	Wild-type	Wild-type
149	Wild-type	Wild-type
150	Wild-type	Wild-type
151	Wild-type	Wild-type
152	Wild-type	Wild-type
153	Wild-type	Wild-type
154	Wild-type	Wild-type
155	Wild-type	Wild-type
156	Wild-type	Wild-type
157	Wild-type	Wild-type
158	Wild-type	Wild-type
159	Wild-type	Wild-type
160	Wild-type	Wild-type
161	Wild-type	Wild-type
162	Wild-type	Wild-type
163	Wild-type	Wild-type
164	Wild-type	Wild-type
165	Wild-type	-
166	Wild-type	Wild-type
167	Wild-type	Wild-type
168	Wild-type	Wild-type
169	Wild-type	Wild-type
170	Wild-type	Wild-type
171	Wild-type	Wild-type
172	Wild-type	Wild-type
173	Wild-type	Wild-type
174	Wild-type	Wild-type
175	Wild-type	Wild-type
176	Wild-type	Wild-type
177	Wild-type	Wild-type
178	Wild-type	Wild-type
179	Wild-type	Wild-type
180	Wild-type	Wild-type
181	Wild-type	Wild-type
182	Wild-type	Wild-type
		Wild-type

a Samples that can be retested.

-, undeterminable.

**Table III:** Comparison of KRAS mutation results with methods other than QP or DS for samples that diverged between the two methods.

No.	DS	QProbe	Scorpion-ARMS
64	Wild-type	Codon 12	p.G12D
69	Wild-type	Codon 12	p.G12V
72	Wild-type	Codon 12	p.G12V
73	Wild-type	Codon 12	p.G12V
80	Wild-type	Codon 12	p.G12D
81	Wild-type	Codon 12	p.G12C
82	Wild-type	Codon 12	p.G12D
63	Wild-type	Codon 12	Wild-type
65	Wild-type	Codon 12	Wild-type
79	Wild-type	Codon 12	Wild-type
46	p.G12D	Wild-type	Wild-type
60	p.G13D	Wild-type	

**Table IV:** The range of temperature for peak area to detection of KRAS and BRAF mutation using the QP method.

	The range of temperature for the peak area		
	
	KRAS codon 12/13	KRAS p.G13D	BRAF V600E
wt peak area	69–75°C	47–52°C	40–48°C
mt peak area 1	62–69°C	52–57°C	48–57°C
mt peak area 2	56–64°C	-	-

**Table V:** Criteria of KRAS and BRAF mutation.

KRAS codon 12/13	
Undeterminable	When reagent reactivity^a^ is ≤0.8
With mutation	When mt peak area 1/wt peak area or mt peak area 2/wt peak area is ≥0.1^b^
No mutation	When mt peak area 1/wt peak area or mt peak area 2/wt peak area is <0.1
KRAS p.G13D	
Undeterminable	When reagent reactivity is ≤0.8
With mutation	When mt peak (~54°C) can be seen visually
No mutation	When mt peak (~54°C) cannot be seen visually
BRAF V600E	
Undeterminable	When reagent reactivity is ≤0.8
With mutation	When mt peak area 1/wt peak area is ≤0.1
No mutation	When mt peak area 1/wt peak area is <0.1

a Reagent reactivity = maximum derivative value/maximum fluorescence value: indication of reactivity.

b Area 2 indicates single nucleotide mutation peak and area 3 indicates dinucleotide mutation peak.

**Table VI:** Measurement results of KRAS codon1 2/13 mutation in biopsy samples without DNA extraction.

	QProbe method				
			
KRAS mutation^a^	Wild-type	Codon 12	p.G13D	Undeterminable	Concordance rate^c^
Wild-type	18	0	0	1	94.7% (100.0%)
Codon 12^b^	0	10	0	0	100.0%
p.G13D	0	0	3	0	100.0%
					Total 96.9% (100.0%)

a Results from Direct Sequence method.

b Codon 12/13 mutations except p.G13D.

c Numbers in parenthesis exclude samples with no results.
